# Serum Level of Brain-Derived Neurotrophic Factor and Thrombotic Type Are Predictive of Cognitive Impairment in the Acute Period of Ischemic Strokes Patients

**DOI:** 10.1155/2023/5578850

**Published:** 2023-03-15

**Authors:** Yaroslava Yu. Havlovska, Nataliya V. Lytvynenko, Anastasiia D. Shkodina

**Affiliations:** ^1^Department of Neurological Diseases, Poltava State Medical University, Poltava 36007, Ukraine; ^2^Neurological Department, Municipal Enterprise “1 City Clinical Hospital of Poltava City Council”, Poltava, Ukraine

## Abstract

40–70% of patients after a stroke, including a mild one, may experience cognitive impairment. Brain-derived neurotrophic factor (BDNF) plays a significant role in the pathogenesis and rehabilitation of ischemic stroke and also affects the patients' recovery prognosis. An association between cognitive impairment in the poststroke period and lower peripheral BDNF levels is known, but the prognostic significance of serum BDNF levels and clinical characteristics for the risk of developing cognitive impairment in the acute period remains uncertain. We conducted a prospective cohort study of patients in the acute phase of ischemic stroke. Clinical examination, assessment of neurological status, neuropsychological testing, and laboratory analyzes were performed on patients at 1 and 14 days after ischemic stroke. The state of cognitive functions was assessed by the Mini-Mental State Examination scale. Quantification of BDNF in blood serum was performed by solid-phaseenzyme-linked immunosorbent assay (ELISA). We found that within 14 days after an acute ischemic stroke, we found a decrease in the clinical severity of patients compared to 1 day of the onset of the disease before the start of treatment and a significant decrease in the level of BDNF in the blood serum of patients with ischemic stroke both on the first and on the 14th day. However, during the 2 weeks of the acute period, no significant changes were detected, despite the general improvement of the clinical condition. In our study, cognitive impairment was found in almost half of the patients on the first day of ischemic stroke, and there was no significant reduction in this prevalence over 2 weeks. We found that a low level of BDNF and a thrombotic subtype of ischemic stroke can be risk factors for cognitive impairment in the acute period, which can be useful in planning treatment and rehabilitation measures.

## 1. Introduction

On a global scale, the prevalence of stroke is from 100 to 300 cases per 100,000 population, that is, about 16 million cases per year. The problem remains acute. The relevance of the problem of cerebral stroke is due to the high frequency of the disease, its serious consequences, and insufficient effectiveness of treatment. Stroke accounts for 5.7 million deaths annually worldwide, second only to coronary heart disease as a cause of death [1]. In Ukraine, there are about 300 cases of ischemic stroke (IS) per 100 thousand population, which exceeds the average frequency of cases in economically developed European countries (200 per 100 thousand population). Along with this, according to official statistics, about 40% of stroke patients die every year in Ukraine [2].

Cognitive disorders can be a symptom of many central nervous system diseases, both acute and chronically progressive. It is known that many vascular diseases are accompanied by changes in the mental state, especially in old age [3, 4]. In 40–70% of patients after a stroke, including a mild one, cognitive impairment may occur [5]. The highest risk of their development is noted in the first 6 months, and after 12 months, the total number of patients with poststroke cognitive disorders increases. The results of the study with the longest followup period showed that within 25 years after the stroke, dementia developed in almost half of the patients. In addition, new evidence has been obtained that motor dysfunction can also cause the occurrence of cognitive disorders and their progression [6].

Degenerative processes in IS begin almost instantly and the first neurons die within a few minutes creating the nucleus of a stroke, which is the acute phase of a stroke. The lack of oxygen initiates the ischemic cascade: a series of biological processes that lead to cell death, damage to adjacent cells, and hyperactivation of the immune system. Due to hypoxia, a penumbra where cells are damaged and do not function, but can still regenerate, is formed around the stroke core. That is why it is important to study biomarkers at the beginning of a vascular disaster [7, 8].

Brain-derived neurotrophic factor (BDNF) is a growth factor that affects neuronal maturation, synaptic plasticity, synapse formation, and neurogenesis [9]. BDNF plays a significant role in the pathogenesis and rehabilitation of IS and also affects the prognosis of the patient's recovery in the poststroke period. It is well known that low levels of circulating BDNF are associated with high stroke risk and poor recovery [10, 11]. However, its expression in brain tissues can be dramatically stimulated by the stroke itself. Clinically positive results have been demonstrated with several stroke treatments that manipulate BDNF levels, including administration of hormones and compounds targeting neurotransmitters, stem cell transplantation, and regulation of other related genes [12].

The crucial role of BDNF in the pathogenesis of IS was first revealed by studies focusing on the treatment of aphasia and motor impairments, which are largely mediated by the process of neuroplasticity [13]. An association between cognitive impairment in the poststroke period and lower peripheral levels of BDNF has also been found, which may be related to impaired signalling [14]. However, the prognostic significance of serum BDNF level and clinical characteristics regarding the risk of developing cognitive impairment in the acute period of ischemic stroke remains uncertain. Our hypothesis is that these indicators can be predictors of the development of cognitive disorders in the acute period of ischemic stroke, but the degree of their influence and the possibility of use are unknown.

## 2. Materials and Methods

### 2.1. Study Design

We conducted a prospective cohort study of patients in the acute phase of ischemic stroke. Patients underwent clinical examination, assessment of neurological status, neuropsychological examination, and laboratory tests at 1 and 14 days after IS.

### 2.2. Setting

The study was conducted in neurological departments of local regional and city hospitals in Ukraine during 2020-2021. On the first day after an ischemic stroke, patients were offered participation in the study after receiving emergency care and were informed about the purpose and procedures to be carried out. All patients included in the study gave informed consent for participation. The research protocol was approved by the local bioethics committee of the university and was conducted in accordance with the Helsinki Declaration of the World Medical Organization. During the first visit on the first day, a comprehensive clinical examination was also performed, neurological status was assessed, stroke severity was determined, brain magnetic resonance imaging or computer tomography was performed, neck vascular ultrasound and cardiac ultrasound were performed to determine the subtype of ischemic stroke, cognitive function was assessed, and blood was collected for determining the level of BDNF. On the 14th day after the patient's ischemic stroke, the above-given procedures were repeated. To determine the degree of severity of symptoms in the general population, a control group of individuals who underwent inpatient treatment in the neurological department but did not have cerebrovascular pathology, was recruited.

### 2.3. Participants

The study included 89 patients of neurological departments, of whom 59 had ischemic stroke and 30 did not have cerebrovascular pathology. During the screening, the patient's compliance with the following inclusion criteria was determined:

- Mild and moderate severity of stroke according to the National Institutes of Health Stroke Scale (NIHSS < 14 points)
- Clear consciousness or a state of mild stupor (Glasgow coma scale 13–15 points)
- Atherothrombotic or cardioembolic subtype IS according to the TOAST classification
- Ischemic stroke in the carotid basin

Exclusion criteria are as follows:

- Age is less than 18 years and older than 80 years
- The presence of clinical and neuroimaging confirmed intracranial hemorrhage, damage to two or more basins
- The presence of a previously experienced acute violation of cerebral blood circulation according to anamnestic, clinical, and neuroimaging
- Severe condition of the patient (sopor or coma at the time of hospitalization)
- Cryptogenic, lacunar, or hemodynamic subtypes IS
- Aphasia
- Chronic vascular lesions of the brain
- Strokes in history
- Neurodegenerative or mental diseases in the anamnesis

### 2.4. Variables

The diagnosis was established taking into account the current International Classification of Diseases 10th revision. Examination and treatment of patients were carried out in accordance with the unified clinical protocol for the provision of medical care assistance to patients with ischemic stroke (Order of the Ministry of Health of Ukraine No. 602 dated August 3, 2012). We also performed a clinical assessment of stroke severity, its subtype, and cognitive function assessment and measured the level of BDNF in the blood serum of all participants.

### 2.5. Biases

To prevent selection bias, the selection of patients with and without ischemic stroke was performed in different clinics in parallel during a defined period, based on inclusion/exclusion criteria and patient consent. Classification bias was prevented by standardizing measurements across all study participants using the same instruments. We performed pseudorandomization by age and gender in order to prevent confounding bias.

### 2.6. Study Size

To calculate the sample size, we used the riskcalc.org online tool. We considered the level of cognitive functions to be the main primary outcome that we studied. According to a study by Pushko, the average value of the Mini-Mental State Examination (MMSE) scale in patients with ischemic stroke in the acute phase in the Ukrainian population is 22.5 points, and in healthy representative individuals, it is 28.3 [15]. We assume that the standard deviation in the population will be 9 points. Thus, with a type I error probability of 0.05, a power of 80%, and a ratio of patients in the stroke versus control group of 2 : 1, the minimum sample size would be 86 patients, including 57 with ischemic stroke and 29 controls.

### 2.7. Stroke Severity Assessment

Stroke severity was determined using the NIHSS scale. According to NIHSS results, up to 5 points were considered as mild stroke, 6–13 points as moderate stroke, 14–20 points as severe stroke, and more than 20 points as very severe stroke.

### 2.8. Cognitive Function Assessment

The state of cognitive functions of the examined patients was studied according to the MMSE scale, according to which 28–30 points were assessed as the absence of cognitive impairment, 25–27 points as predemented cognitive impairment, 20–24 points as mild dementia, 11–19 points as moderate dementia, and 10 or less points as severe dementia. For binary analysis, the presence of cognitive disorders was determined by the sum of points less than 25.

### 2.9. Measurement of BDNF Level in Serum

Quantitative determination of BDNF in blood serum was performed on the basis of the Research Institute of Genetic and Immunological Foundations of Development of Pathology and Pharmacogenetics of the Poltava State Medical University by solid-phaseenzyme-linked immunosorbent assay (ELISA) using the brain-derived neurotrophic factor (BDNF) kit (Cloud-Clone Corp., USA). The study was conducted in dynamics twice: the first blood sample was taken from the patient on the 1st day of occurrence of IS and the second blood sample was taken on the 14th day after IS. Peripheral venous blood was collected under sterile conditions from the ulnar vein using a disposable sterile vacuum blood collection system with a coagulation activator (BD Vacutainer®, Great Britain). To obtain serum, blood samples were kept for 30 minutes after collection at room temperature for coagulation. Then, the blood samples were centrifuged at a speed of 3000 rpm for 10–15 minutes at room temperature. Blood serum obtained in this way was stored at a temperature of −80°C until the analysis.

### 2.10. Ethics Approval

Approval was obtained from the ethics committee of Poltava State Medical University (protocol No. 178, 24th of December 2019). The procedures used in this study adhere to the tenets of the Declaration of Helsinki. Informed consent was obtained from all individual participants included in the study.

### 2.11. Statistical Methods

Statistical analysis was performed using IBM SPSS Statistics 26.0 and MS Excel 2019. Pseudorandomization was performed to standardize patient groups by age and sex. Quantitative values are presented in the form of the arithmetic mean (M) and standard deviation (SD) and categorical values as absolute (abs.) and relative (%) values. A Mann–Whitney test for independent or Wilcoxon test for dependent groups was used to compare quantitative variables between two groups, according to the type of distribution analyzed by Shapiro–Wilk test. A comparison of independent categorical variables was performed by calculating *χ*^2^. The McNemar test was used to analyze dynamic changes in quality indicators. The influence of factors on a binary variable was assessed by performing a binary logistic regression analysis followed by the calculation of the odds ratio (OR) and 95% confidence interval (95% CI). The coefficients of the predictive model are presented in the form of value (*B*) and standard error (*m*). To check the consistency of the model, the Hosmer–Lemeshow test was used, and the degree of variance described by the constructed model was evaluated by Nagelkerke's pseudo-*R*^2^. To determine the quality of the built model, ROC analysis was performed by determining the area under the curve (AUC). The selection of the optimal limit value of the test was carried out using the Youden Index calculation method. To characterize the model, indicators of specificity, accuracy, and sensitivity were calculated. The limit value of *p* was considered to be 0.05.

## 3. Results

**Figure 1:**
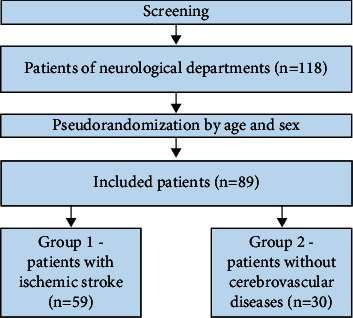
Study selection process. The authors screened 118 patients in neurological departments who met the criteria listed as follows. After pseudorandomization, the authors include 89 patients in this study. Group 1 consists of 59 patients with ischemic stroke. Group 2 contains 30 patients without cerebrovascular pathology.

We screened patients who were being treated in the neurological department. The selection process is presented (see Figure 1).

The demographic and clinical characteristics of the study population are presented (see Table 1). After pseudorandomization, no statistically significant differences were found between groups by age and gender.

In group 1, patients with an atherothrombotic subtype of ischemic stroke prevailed over cardioembolic stroke (76.3% vs. 23.7%). During 14 days of treatment of the acute period of ischemic stroke, a statistically significant decrease in the score on the NIHSS scale was established (*р*=0.038).

**Figure 2:**
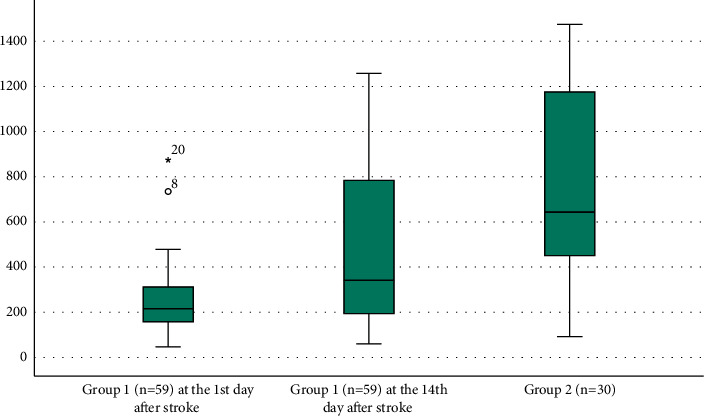
BDNF serum levels in patients with ischemic stroke at the 1st and 14th days of the acute period in comparison with the control group.

The levels of BDNF in our groups are presented (see Figure 2).

Statistically lower indicators of the BDNF level were established on the 1st day of ischemic stroke in group 1 compared to group 2 (*р*=0.029). At the same time, no statistically significant changes in BDNF concentration were found within 14 days after ischemic stroke (*p*=0.503). The frequency of cognitive disorders on the 1st day of ischemic stroke was statistically significantly higher in group 1 compared to group 2 (*p*=0.006); however, within 14 days after ischemic stroke, no significant changes were found in the number of patients with cognitive disorders in group 1 (*p*=0.468).

To assess the risk factors for cognitive impairment in the acute period of ischemic stroke, we performed a binary logistic regression analysis using the type of ischemic stroke, BDNF concentration, and NIHSS 1st day score as predictors. When the specified parameters are included, the model is assembled in 3 steps. The inverse Wald method was used to select 2-factor characteristics with a statistically significant effect: BDNF concentration for 1 day (*X*1) and atherothrombotic subtype of ischemic stroke (*X*2). It is demonstrated estimates of the coefficients of the regression model for cognitive disorders (Table 2).

It was demonstrated that an increase in BDNF level leads to a decrease in the risk of cognitive impairment (OR = 0.98, 95% CI 0.98–0.99, *p*=0.010) and an atherothrombotic subtype of ischemic stroke increases it (OR = 10.48, 95% CI 1.53–71.69, *p*=0.017). To check the consistency of the model, the Hosmer–Lemeshow test was used (*χ*2 = 8.98, d*f* = 8, *p*=0.345), which confirms the correspondence between the data predicted by the model and the real data. According to Nagelkerke's pseudo-*R*^2^ value, the factors included in the model explained 51.7% of the variance of the dependent variable.

**Figure 3:**
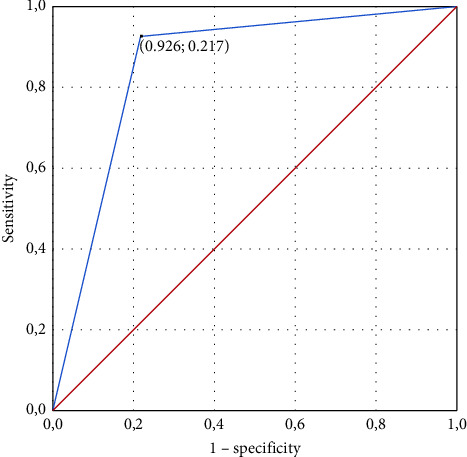
ROC curve for prognostic model of cognitive impairments in acute period of ischemic stroke based on BDNF level on the 1st day and hemodynamic subtype.

To determine the limit value of the probability of the forecast of the proposed model and to evaluate its prognostic characteristics, the method of construction and analysis of the curve of operational characteristics was used. The ROC curve of the test is shown (see Figure 3). The proposed test allows predicting the risk of cognitive impairment in the acute period of ischemic stroke depending on the hemodynamic subtype and BDNF concentration for 1 day (AUC = 0.85 ± 0.06 (95% CI 0.74–0.97), *p* < 0.001).

The selection of the optimal limit value of the test was carried out using the Youden index calculation method. The optimal decision-making limit (DML) was *P*_DML_ = 0.509: for *P*_(patient)_ ≥ P_DML_, it is possible to predict the risk of cognitive disorders and for *P*_(patient)_ < *P*_DML_, it is possible to predict the absence of cognitive disorders. The chosen decision-making threshold determines the value of sensitivity at the level of 92.6%, specificity at 78.3%, and accuracy at 86.1%.

We found the prognostic role of BDNF concentration and hemodynamic subtype in relation to cognitive impairment in the acute period of ischemic stroke.

## 4. Discussion

Cardiovascular diseases are the main cause of disability and mortality in Ukraine [16]. Along with this, stroke is the third most common cause of disability worldwide, which contributes to the development and increasing severity of cognitive impairment. The high incidence of poststroke cognitive impairment calls for the development of effective treatment strategies in which preventive strategies are more attractive, especially those targeting modifiable risk factors [17].

We found that the thrombotic subtype of ischemic stroke predominated among the studied patients, which is consistent with previous studies [18]. It was determined that the frequency of thrombotic stroke can reach 54%, and embolic stroke can range from 10% to 26%. At the same time, a larger share of the thrombotic subtype was found in the Chinese population compared to data from predominantly white populations of European origin [18].

Within 14 days after an acute ischemic stroke, we found a decrease in the clinical severity of patients compared to 1 day after the onset of the disease before the start of treatment. There are few studies examining the condition of patients in the first 2 weeks after a stroke. The evidence for recovery of motor function is limited, but there is evidence for the effectiveness of movement therapy, which may be beneficial if started within the first 2 weeks or intensive therapy in patients with severe aphasia. And, although the optimal time to start rehabilitation after a stroke remains uncertain, accumulated data indicate the benefit of starting rehabilitation programs in the first 14 days but not in the first 24 hours [19]. Such changes may be associated with dynamic changes in the concentration of BDNF, which is known to be involved in neuroplasticity processes. Thus, it was demonstrated that patients who received rehabilitation procedures starting 3–6 days after the onset of a stroke had better motor functions, psychological profile, and cognitive abilities and, along with this, higher levels of BDNF in blood serum 6 months after the onset [20].

We found a significant decrease in the level of BDNF in the blood serum of patients with ischemic stroke both on the first and on the 14th day. However, during the 2 weeks of the acute period, no significant changes were detected, despite the general improvement of the clinical condition. Decreased BDNF concentration in ischemic stroke is well known and confirmed by the latest meta-analysis [21]. A relationship between stroke severity and BDNF levels is commonly reported, but the lack of significant changes in our study in the face of clinical improvement may be due to the fact that only mild and moderate stroke patients were included in the study. It has been reported that BDNF levels may be influenced by baseline NIHSS and the presence of risk factors [22].

Poststroke cognitive disorders of various degrees of severity are found in 40–70% of patients who have suffered a stroke, and this is about half of the patients, including people with a mild stroke. The prevalence of dementia in the first 3–6 months after a stroke ranges from 5 to 32% and after 12 months from 8 to 26%. The highest risk of developing poststroke cognitive disorders is observed during the first months after a stroke, which is probably due to previously unrecognized and undiagnosed initial manifestations of cognitive disorders [23]. In our study, cognitive impairment was found in almost half of the patients on the first day of ischemic stroke, and there was no significant reduction in this prevalence over 2 weeks. Although stroke is known to lead to cognitive decline, we cannot say with certainty that the increased proportion of cognitive impairment in stroke patients compared to controls was solely due to stroke, as it is not possible to rule out prestroke cognitive impairment. Some authors report that 10% of stroke patients had prestroke dementia [24].

Our study found that low BDNF and thrombotic subtype of ischemic stroke may be risk factors for cognitive impairment in the acute poststroke period. An association between higher baseline BDNF levels and a lower rate of cognitive impairment progression over 1 year in Alzheimer's disease has also been reported. BDNF plays a key role in modulating synaptic transmission and plasticity in the brain, important in learning and memory processes. The potential retarding effect of BDNF on the rate of cognitive decline may be a consequence of its neuroprotective effects in the brain [25]. It was also found that cognitive functions in stroke patients are impaired with an increase in the content of *β*-amyloid peptide and a decrease in BDNF, the mechanism of which is associated with a decrease in cyclic adenosine monophosphate and phosphorylated-cAMP-response element binding protein. BDNF protects neurons from apoptosis, a key process that directly leads to neuronal cell death and brain damage after stroke, by activating downstream PI3K/Akt and MAPK/ERK pathways. Neurogenesis is a characteristic of the brain's regeneration potential following damage, and the BDNF-TrkB signalling pathway plays a critical part in these processes [12, 26].

Also, it should be mentioned that the small sample size is an important limitation of our study. However, it was noted in recent studies that it is challenging to identify significant differences when the group size is less than 60 persons and the necessity to validate such data using a western blot, which was not done in this study [27].

It is also known about the prognostic importance of subtypes of ischemic stroke in terms of patient recovery [28]. Atherothrombotic, thrombotic subtype, or large artery atherosclerosis is associated with cerebral angiopathy and recurrent transient ischemic attacks, which can lead to cognitive decline even before the development of a stroke and thus contribute to the risk of cognitive deterioration in the poststroke period [29, 30].

The identified risk factors for cognitive dysfunction in the acute period of ischemic stroke can be useful in planning treatment and rehabilitation measures. However, more in-depth research aimed at studying the pathogenetic relationship between stroke subtypes and the level of cognitive functions is needed. The level of BDNF in the acute period of ischemic stroke can be considered as a potential biomarker for the recovery of not only motor but also cognitive functions, as well as contribute to the search for additional targeted therapy in the course of stroke prevention.

It should be noted among the limitations of our study that we did not include patients with severe ischemic stroke, due to the impossibility of assessing cognitive functions on the first day. In addition, our study was conducted in one region and did not take into account the racial and genetic characteristics of the patients, although it is known that the interaction of the BDNF (rs6265) Val < Met polymorphism with cerebral lesions is associated with cognitive dysfunction and this destructive effect may be exacerbated by damage to the dominant hemisphere [31].

## 5. Conclusions

Thus, our study revealed a relationship between low peripheral BDNF levels and the thrombotic subtype of ischemic stroke with cognitive impairment in the acute period of ischemic stroke. We have developed a prognostic model that allows you to predict the risk of cognitive impairment in the acute period. The use of this and similar models, as well as further research in this direction, may contribute to the development of more individualized management assessments to improve long-term treatment outcomes.

## Figures and Tables

**Table 1:** Demographic and clinical characteristics of studied population.

Demographics	Groups		*p* value
	Group 1 (*n* = 59)	Group 2 (*n* = 30)	
Age, M ± SD	61.19 ± 9.61	57.84 ± 4.89	*p*=0.392
Sex, abs. (%)			
M	37 (62.7%)	21 (70.0%)	*χ* ^2^ = 0.46 d*f* = 2 *p*=0.495
F	22 (37.3%)	9 (30.0%)	
Clinical type of stroke abs. (%)			
Thrombotic	45 (76.3%)	—	—
Embolic	14 (23.7%)	—	
NIHSS, M ± SD			
1st day	7.02 ± 3.88	—	—
14th day	4.42 ± 3.39	—	—
*p* value	0.038	—	
BDNF level (pg/ml), M ± SD			
1st day	307.87 ± 245.44	734.65 ± 417.53	*p*=0.029
14th day	470.94 ± 376.17	—	
*p* value	0.503	—	
Cognitive impairment abs. (%)			
1st day	32 (54.2%)	7 (23.3%)	*χ* ^2^ = 7.12, d*f* = 1, *p*=0.006
14th day	28 (47.6%)	—	
*p* value	Mac–Nemar's *χ*^2^ = 0.624, df = 1, *р*=0.468	—	

**Table 2:** Scores for factors ranked by the strength of influence on the presence of cognitive disorders in the acute period of ischemic stroke in ascending order.

No.	Factor	Category	*B* ± *m*	OR	95% CІ	*p* value
1	Constant		0.81 ± 1.09	—	—	0.460
2	*X*1	BDNF on the 1st day (pg/ml)	−0.009 ± 0.004	0.981	0.984–0.998	0.010
3	*X*2	Thrombotic subtype (yes)	2.35 ± 0.98	10.48	11.53–71.69	0.017
		Thrombotic subtype (no)	0	—	—	—

## Data Availability

The data used in the study are available upon request.

## Abbreviations

Akt: Protein kinase B
AUC: Area under the curve
BDNF: Brain-derived neurotrophic factor
cAMP: Cyclic adenosine monophosphate
CI: Confidence interval
DML: Decision-making limit
ELISA: Enzyme-linked immunosorbent assay
ERK: Extracellular signal-regulated kinases
IS: Ischemic stroke
MAPK: Mitogen-activated protein kinase
MMSE: Mini-Mental State Examination
NIHSS: National Institutes of Health Stroke Scale
OR: Odds ratio
PI3K: Phosphoinositide 3-kinase
SD: Standard deviation
TrkB: Tropomyosin receptor kinase B.
